# Removal of an endometrioma passing through the left greater sciatic foramen using a concomitant laparoscopic and transgluteal approach: case report

**DOI:** 10.1186/s12905-019-0796-0

**Published:** 2019-07-12

**Authors:** Liang Yanchun, Zhao Yunhe, Xia Meng, Chen Shuqin, Zhu Qingtang, Yao Shuzhong

**Affiliations:** 1 Department of Obstetrics and Gynecology, The First Affiliated Hospital, Sun Yat-sen University, No. 58, the 2nd Zhongshan Road, Yuexiu District, Guangzhou, 510080 Guangdong Province China; 2 Department of Orthopedic Trauma & Microsurgery, The First Affiliated Hospital, Sun Yat-sen University, Guangzhou, 510080 China

**Keywords:** Endometriosis, Laparoscopy, Minimally invasive surgery, Sciatic nerve, Transgluteal surgery, Case report

## Abstract

**Background:**

The combination of intrapelvic and extrapelvic endometriosis is a very rare condition in gynecology. Patients with endometriosis involving the sciatic nerve are easily misdiagnosed because they usually present with atypical symptoms of endometriosis. Here, we present a rare case of an endometrioma passing through the left greater sciatic foramen. Removal of the endometriotic lesion was performed with a concomitant laparoscopic and transgluteal approach through the cooperation of gynecologists and orthopedic (neuro)surgeons.

**Case presentation:**

A 20-year-old woman presented with complaints of severe dysmenorrhea lasting for more than 6 years and dysfunction of her left lower limb lasting for approximately 4 months. Both CT and MRI demonstrated a suspected intrapelvic and extrapelvic endometriotic cyst (7.3 cm × 8.1 cm × 6.5 cm) passing through the left greater sciatic foramen. Laparoscopic exploration showed a cyst full of dark fluid occupying the left obturator fossa and extending outside the pelvis. A novel combination of transgluteal laparoscopy was performed for complete resection of the cyst and decompression of the sciatic nerve. Postoperative pathology confirmed the diagnosis of endometriosis. Long-term follow-up observation showed persistent pain relief and lower limb function recovery in the patient.

**Discussion and conclusions:**

When a woman complains of unexplained unilateral sciatica, especially a woman suffering from dysmenorrhea, endometriosis of the sciatica nerve should be considered as a potential etiology. Complete excision of the endometriotic lesion and adequate neurolysis (or decompression) of the sciatic nerve through the multidisciplinary cooperation of experienced gynecologists with proper training in laparoscopic pelvic (neuro)surgery and orthopedic (neuro)surgeons is effective.

## Background

Endometriosis is classically defined as the presence of endometrial glands and stroma outside the uterine cavity [1]. Intrapelvic endometriosis is a fairly common condition found in women of reproductive age, usually with typical symptoms of dysmenorrhea or chronic pelvic pain. It is usually not difficult to diagnose and many treatment strategies (including drugs or surgical techniques) have been developed to manage intrapelvic endometriosis. Therefore, rare cases of extrapelvic endometriosis seem to be greater challenges for gynecologists. Endometriosis involving the muscles and nerves within a buttock are usually misdiagnosed by orthopedic surgeons due to its typical symptoms of swelling, articular dyskinesia and sensory disturbance of the unilateral buttock as well as the lower limb [2].

Moreover, extension of endometriosis from within the pelvis to outside the pelvis is very rare. Here, the authors describe the diagnosis and treatment of a 20-year-old woman with an endometriotic cyst exiting the pelvis through the left greater sciatic foramen. More importantly, the surgical team in this case developed a novel technique combining a transabdominal and transgluteal approach to completely excise the lesion. Postoperatively the patient recovered without sequelae.

## Case presentation

The study received approval from the Ethics Committee of The First Affiliated Hospital, Sun Yat-Sen University. Informed consent was obtained from the patient.

**Fig. 1:**
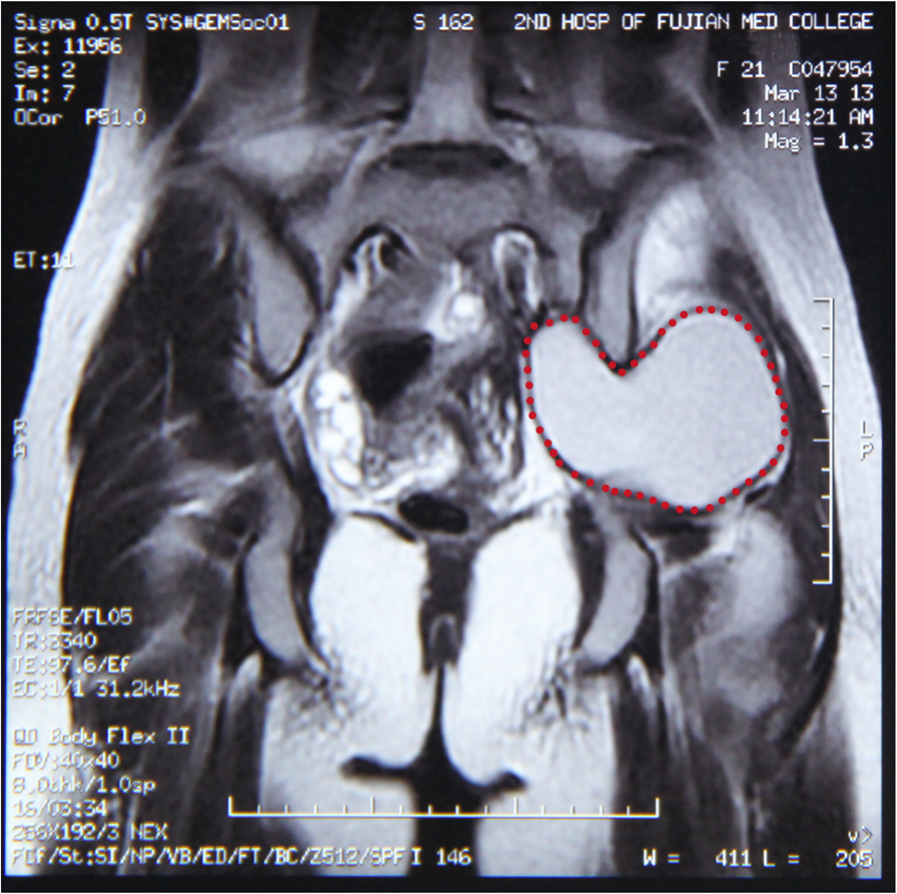
T2-weighted MRI image of the lesion. A cyst full of fluid was observed in the pelvis and extended to the left buttock through the greater sciatic foramen (pink dotted circle; heart-shaped area)

A 20-year-old woman (G0P0) presented with severe dysmenorrhea lasting for more than 6 years. In the beginning, the pain started from the first day of her menstruation and disappeared after menstruation ended. One year before presentation, it became increasingly intense, lasting longer than before (more than 1 week) and mainly limited to the left lower abdomen. Four months before presentation, she complained of occasional pain and numbness in the left lower limb, especially the popliteal fossa and the sacroiliac joint. However, the pain was not associated with her menstruation. Then, she visited orthopedic surgeons and underwent computed tomography (CT) and magnetic resonance imaging (MRI) of the pelvis as well as the buttocks. CT showed uterus didelphis as well as a cyst in the left buttock (7.3 cm × 8.1 cm × 6.5 cm). MRI confirmed that there was a cyst full of fluid located in the pelvis and extending into the left buttock through the greater sciatic foramen (Fig. 1). Both of the imaging examinations failed to provide a definite diagnosis of the cyst (especially whether it was benign or malignant), which confused the orthopedic surgeons. To elucidate the diagnosis of the cyst, a needle aspiration of the cyst was performed. Pathology revealed many red blood cells with the infiltration of a few neutrophils, macrophages, lymphocytes and epidermal cells, but without any malignant cells.

Since the diagnosis was still unclear, the orthopedic surgeons suggested that the patient consult with gynecologists at the same time. Before she came to our clinic, the pain continued to progress. She did not receive any treatments (either oral contraceptives or GnRHas) to alleviate her pain. The adduction function of her left hip joint was severely affected, and she could not even lift her left limb. Bimanual pelvic exploration revealed a mass in the left pelvis, with tenderness on compression of the left buttock. Transvaginal ultrasound at our hospital showed uterus didelphis, with a hematometra in the left uterus. A cyst with a homogenous dark area of liquid (9.3 cm × 6.6 cm) and a clear margin was also detected in the left pelvis. Ultrasound examination also showed that she had only one kidney. The serum level of CA125 was 87.20 U/mL.

A clinically suspected diagnosis of an endometriotic cyst passing through the left greater sciatic foramen was established. A surgical team composed of gynecologists, orthopedic surgeons and microsurgical experts discussed different treatment strategies for the case in detail. Finally, a novel minimally invasive technique combining transabdominal and transgluteal laparoscopy to remove the cyst was proposed. This tentative surgical plan with a transgluteal open approach as an alternative was also accepted by the patient.

**Fig. 2:**
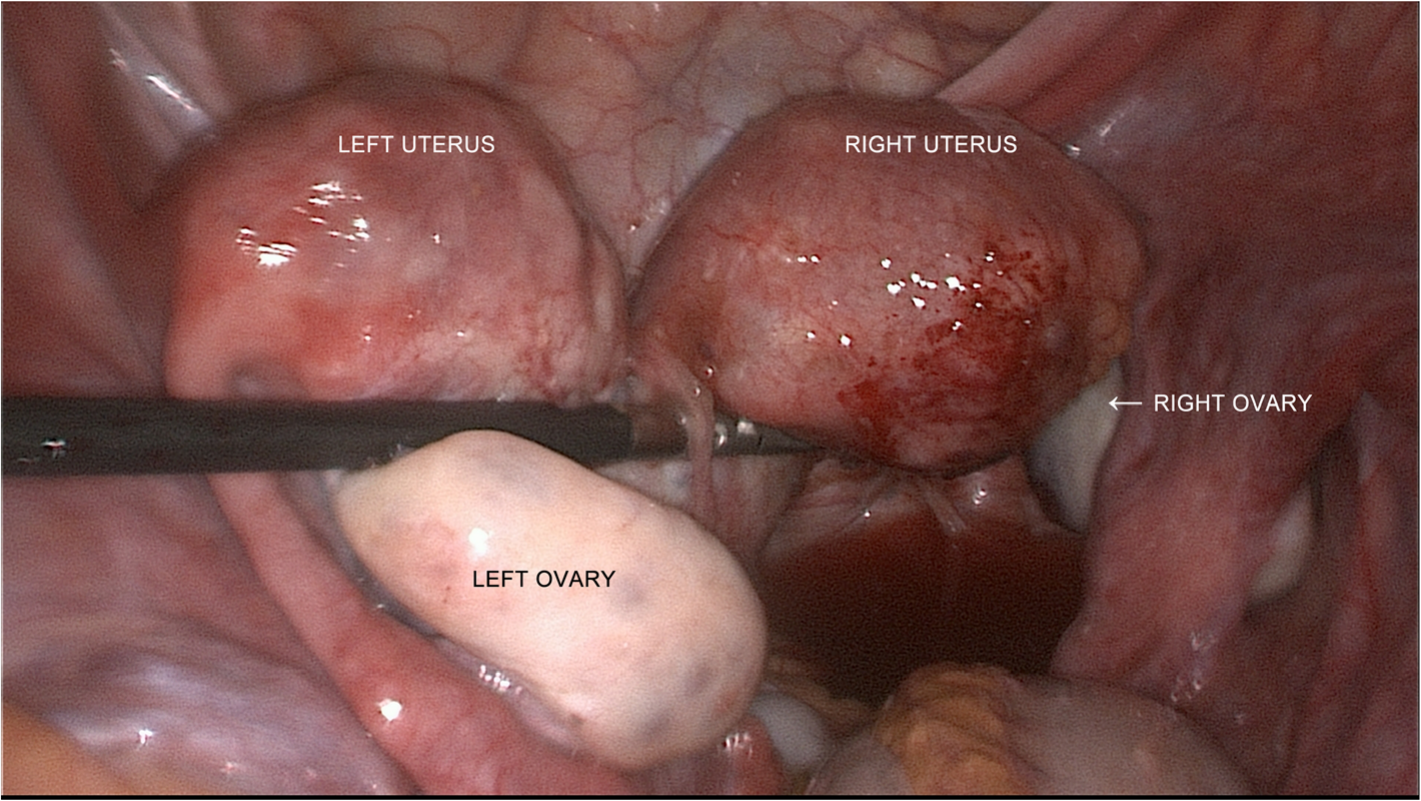
Laparoscopic exploration of the pelvis

**Fig. 3:**
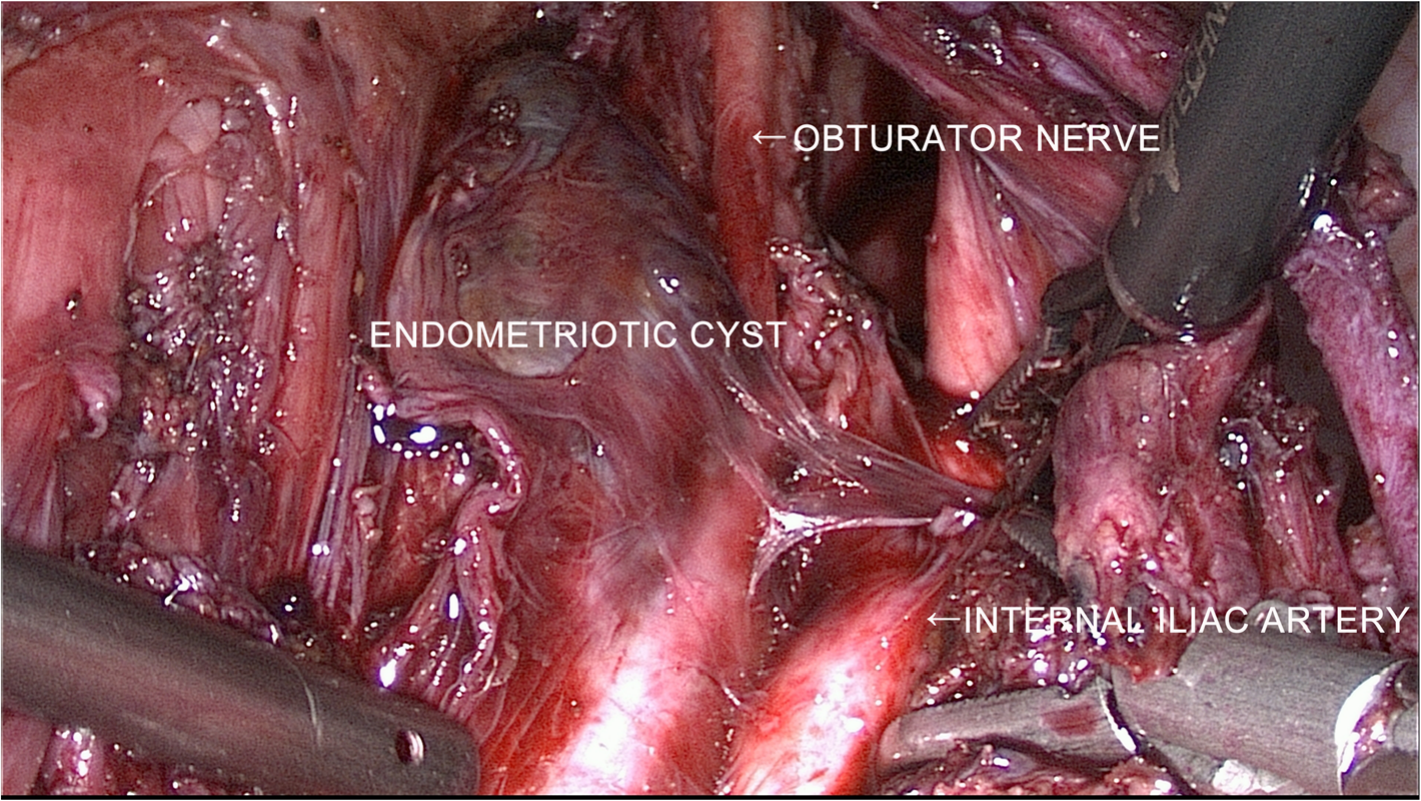
Laparoscopic image of the endometriotic cyst in the left obturator fossa

**Fig. 4:**
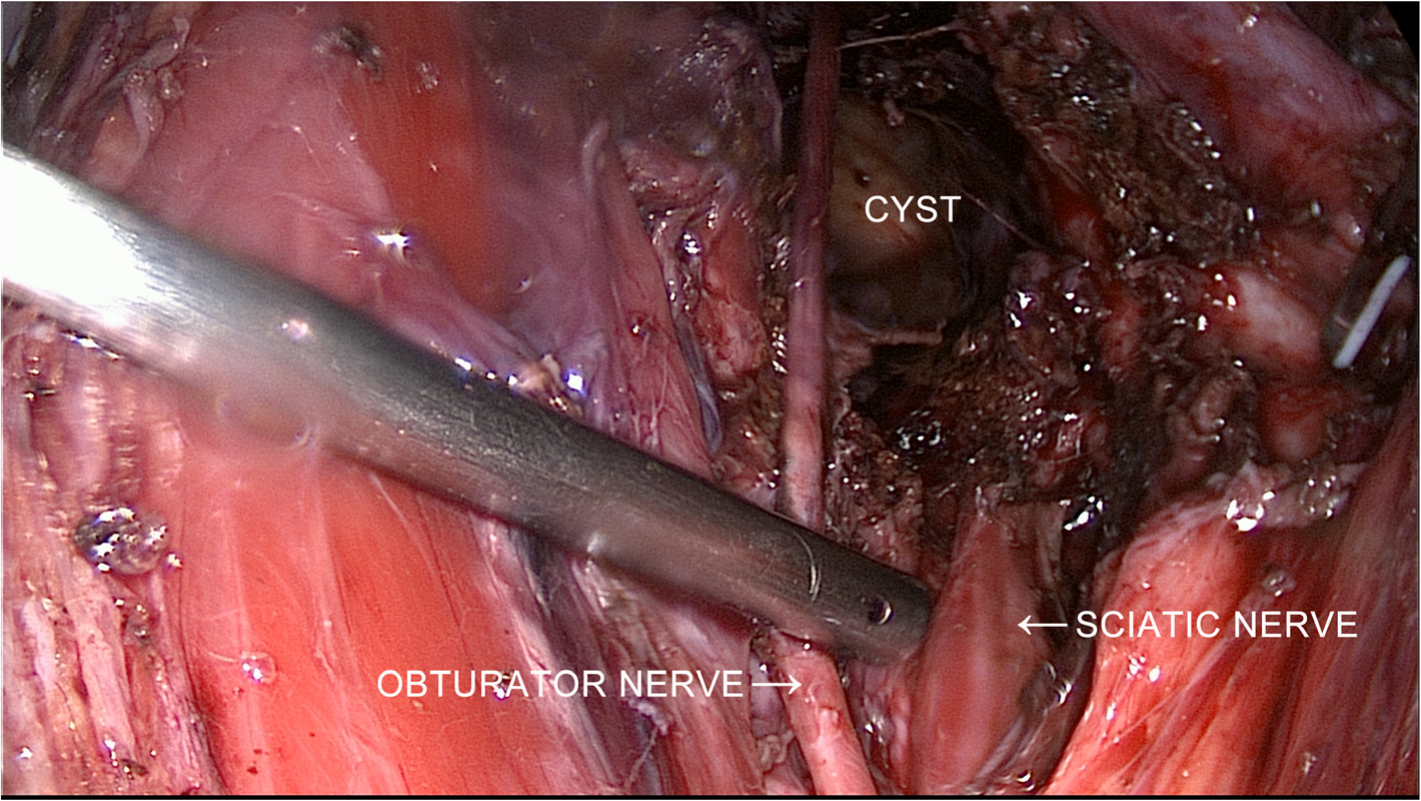
Laparoscopic image of the topological relationship of the endometriotic cyst, the left obturator nerve and the sciatica nerve

Laparoscopic surgery was performed by the gynecologists first. Didelphis uterus was found, with the left adnexa attached to the left uterus, and the right adnexa attached to the right uterus (Fig. 2). The left infundibulopelvic ligament, left external iliac artery and vein, and left internal artery were dissected, finally exposing the left obturator fossa. We found a purple cyst between the left obturator nerve and left psoas muscle (Fig. 3). When separating the cyst and left obturator nerve, the cyst was perforated, and a large volume of dark fluid flowed out from the cyst. After we cleared the fluid from inside the cyst, we found that the cyst had a smooth internal surface, further supporting the preoperative diagnosis of endometrioma. We carefully dissected the cyst along the margin until it exited the pelvis through the left greater sciatic foramen. The pelvic part of the left sciatic nerve was exposed laparoscopically because it was also compressed by the cyst (Fig. 4).

**Fig. 5:**
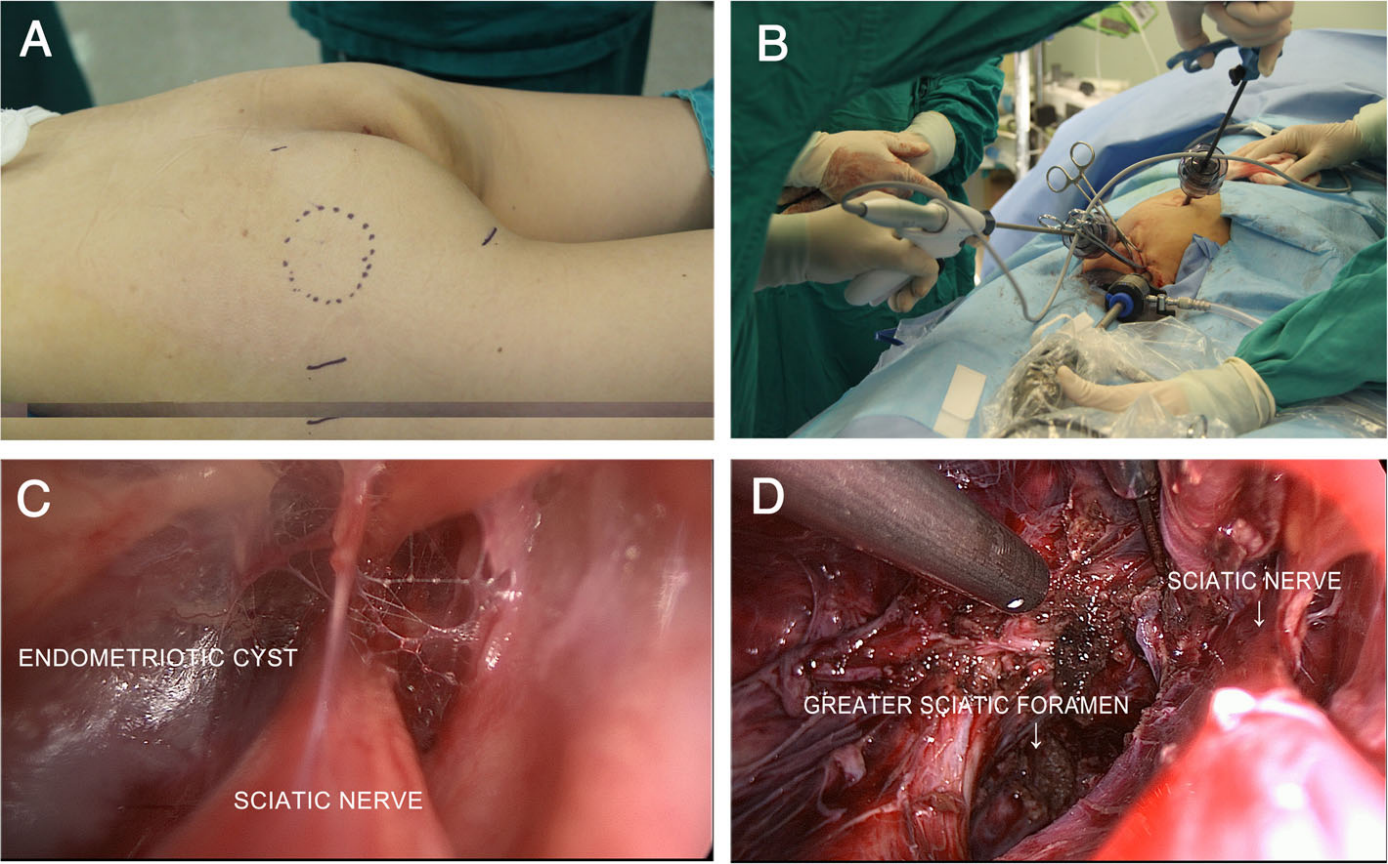
Images of transgluteal laparoscopic surgery. **a** Selection of the incision point on the left buttock. **b** Placement of the laparoscopic trocars. **c** Exposure of the extrapelvic part of the endometriotic cyst near the left sciatic nerve. **d** Complete resection of the endometriotic cyst and decompression of the left sciatic nerve

The patient was then placed in a ventral decubitus position, to continue the transgluteal laparoscopic surgery. The reflection point of the left sciatic nerve on the skin of the left buttock was located first. Three apexes of a triangle around this point were selected for the insertion of laparoscopic trocars (Fig. 5 a and b). A straight incision of approximately 4 cm was created posteromedical to the left buttock. The left sciatic nerve was exposed first. Then the laparoscope was inserted through the incision. We dissected along the space between the left sciatic nerve and the surrounding muscles using an ultrasonic scalpel and clamps under guidance of the laparoscope, until we reached the endometriotic cyst (Fig. 5c). We separated and removed the cyst from the tissues to which it was attached (including the left sciatic nerve, left piriformis muscle, sacrospinous ligament and ischium) (Fig. 5d). Finally, it was completely removed through the longer incision in the left buttock. Pathological examination confirmed the endometriotic nature of the cyst.

Obvious pain relief in the left lower limb was achieved 3 weeks after the operation. The movement disorder of the left lower limb completely recovered after 6 months, with continuous regular rehabilitation exercises. The patient became pregnant (right uterus) successfully right after 6 months of GnRHa treatment postoperatively and gave birth to a healthy baby. The 5-year follow-up examination showed that she continued to be asymptomatic without pain and with normal lower limb function.

## Discussion

Endometriosis is a rare cause of functional disturbance of the lower limb(s). Endometriosis infiltrating the sciatic nerve often causes cyclical sciatica. Since Schlicke reported the first case of endometriosis-induced sciatica in 1946 [3], more than 70 cases have been reported. Most of the cases have had histopathological evidence of infiltration of the sciatic nerve. However, the case reported here only involved an endometriotic cyst compressing the sciatic nerve from the left obturator fossa all the way outside the pelvis through the left greater sciatic foramen. To the best of our knowledge, there no similar cases have been reported in the literature.

Women with symptoms of typical sciatica and/or of unilateral lower limb dysfunction usually visit the orthopedic clinic first. However, when women complain of dysmenorrhea simultaneously, especially when the sciatica is also cyclical and occurs during menstrual periods, orthopedic surgeons should consider endometriosis involving the sciatic nerve. In our case, the pain the patient experienced was occasional at the very beginning of the formation of the endometriotic cyst. When cyclical bleeding of the endometrial tissue inside the cyst occurred and the fluid accumulated gradually, the cyst became increasingly larger, compressing the sciatic nerve progressively. As a result, the pain became increasingly intense, with a pain-free interval that gradually shortened until, after a few months, it became persistent. Fortunately, she was introduced to our department promptly once the imaging examinations revealed a cyst passing through the greater sciatic foramen. More importantly, proper treatment was performed, avoiding permanent damage to the left sciatic nerve.

The pathophysiology of endometriosis is still poorly understood but appears to be multifactorial. The “pocket-sign” theory was proposed and has been accepted by some scholars based on Sampson’s retrograde theory [4, 5]. It is believed that there exists a peritoneal diverticulum permitting ectopic endometrial tissue to migrate to the sciatic nerve. Since the rectosigmoid colon is located in the left pelvis, it forms a natural barrier to protect the left pelvic organs and tissues from infiltration by endometrial cells. As a consequence, right side involvement of the sciatic nerve is more frequently reported. However, in this case, the endometriotic cyst was located on the left side, suggesting that other potential pathogenic mechanisms may be more important. A hematometra in the left uterus was found before the surgery, suggesting that retrograde menstruation through the left fallopian tube may have occurred. No other endometriotic lesions were found in the rest of the pelvis. Therefore, we can exclude spreading of the disease via deepening from the Pouch of Douglas or pelvic peritoneum. This case could confirm the Halban “lymphatic and vascular metastasis” theory, or the Possover “neural theory,” according to which endometriosis could arise in the retroperitoneum from the lymphatic, neural, and hematogenous dissemination of endometrial cells [6, 7].

Vascular damage (such as minor injury or surgery) could lead to hematologic migration of endometriosis, especially in patients without other sites of endometriosis. Menstrual blood reflux is the incentive of endometriosis. The retrograde endometrial debris penetrates neighboring tissues and organs every month, where it triggers locally inflammatory and immunological reaction. Patient with sciatic nerve endometriosis usually suffer from periodic sciatica, sometimes beginning 1 to 2 days before or after the first day of a period. The pain is intense and has a pain-free interval in the beginning. But, as time goes on, it can be progressive and becomes permanent finally.

Clearly, endometriosis afflicts women in different ways in terms of the type and severity of symptoms, how badly the pelvis is affected, and the effect on health and quality of life. Therefore, treatment must be individually selected for each woman, taking other factors such as age, response to previous treatment, surgical complication rates, and the desire for pregnancy into account. In this case, achieving complete removal of the endometriotic cyst and relief of compression of the left sciatic nerve were of equal importance. Both the gynecologists and orthopedic surgeons thought that surgical removal of the cyst was necessary, but selection of the surgical approach seemed to be a difficult challenge. Laparoscopic resection of the intrapelvic part of the cyst and neurolysis of the sciatic nerve could be achieved by gynecologists who were well-trained and familiar with pelvic (neuro)anatomy [8]. The traditional approach to expose the sciatic nerve is transgluteal open surgery; however, the incision is long, and the incidence of perioperative complications is relatively high. Based on knowledge of and skill in extraperitoneal laparoscopic ilioinguinal lymphadenectomy, we proposed a bold and creative attempt based on transgluteal laparoscopy to resect the extrapelvic part of the cyst along the space between the sciatic nerve and the surrounding tissues. Although it was difficult and risky, we succeeded. No intraoperative or postoperative complications occurred in this case. The combined technique of transabdominal and transgluteal laparoscopy was proven to be feasible. The short-term and long-term follow-up observations of the patient showed rapid recovery from the surgery, a low incidence of perioperative complications, and most importantly, a good treatment effect (persistent pain relief and functional recovery of the lower limb).

When a woman complains of unexplained unilateral sciatica, especially a woman also suffering from dysmenorrhea, endometriosis of the sciatica nerve should be considered a potential etiology. Patients are commonly misdiagnosed because they are often sent to orthopedic surgeons, neurologists or neurosurgeons. CT, MRI and transvaginal/transrectal ultrasound are important for the diagnosis. Complete excision of the endometriotic lesion and adequate neurolysis (or decompression) of the sciatic nerve are extremely important. The multidisciplinary cooperation of experienced gynecologists possessing proper training in laparoscopic pelvic (neuro)surgery and orthopedic (neuro)surgeons should gain interest within the medical field as this condition affects more patients than we realize.

## Data Availability

The datasets used and/or analyzed during the current study are available from the corresponding author on reasonable request.

## Abbreviations

CT: Computed tomography
MRI: Magnetic resonance imaging
